# Efficient Modeling of Water Adsorption in MOFs Using Interpolated Transition Matrix Monte Carlo

**DOI:** 10.1021/acsami.4c02616

**Published:** 2024-05-06

**Authors:** Bartosz Mazur, Lucyna Firlej, Bogdan Kuchta

**Affiliations:** † Department of Micro, Nano, and Bioprocess Engineering, Faculty of Chemistry, Wroclaw University of Science and Technology, Wroclaw 50-370, Poland; ‡ Laboratoire Charles Coulomb (L2C), Universite de Montpellier - CNRS, Montpellier 34095, France; § MADIREL, CNRS, Aix-Marseille University, Marseille 13013, France

**Keywords:** water adsorption, atmospheric water harvesting, computational screening, molecular modeling, metal−organic frameworks, transition matrix Monte Carlo

## Abstract

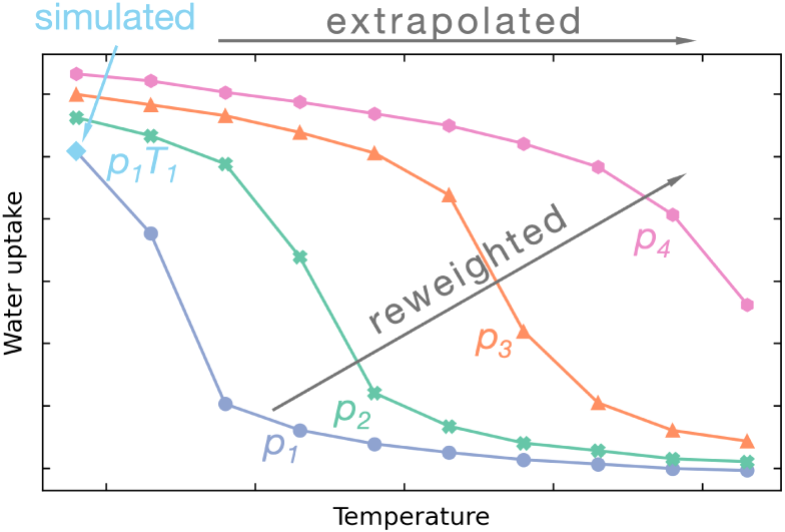

With the specter of accelerating climate change, securing access to potable water has become a critical global challenge. Atmospheric water harvesting (AWH) through metal–organic frameworks (MOFs) emerges as one of the promising solutions. The standard numerical methods applied for rapid and efficient screening for optimal sorbents face significant limitations in the case of water adsorption (slow convergence and inability to overcome high energy barriers). To address these challenges, we employed grand canonical transition matrix Monte Carlo (GC-TMMC) methodology and proposed an efficient interpolation scheme that significantly reduces the number of required simulations while maintaining accuracy of the results. Through the example of water adsorption in three MOFs: MOF-303, MOF-LA2–1, and NU-1000, we show that the extrapolation of the free energy landscape allows for prediction of the adsorption properties over a continuous range of pressure and temperature. This innovative and versatile method provides rich thermodynamic information, enabling rapid, large-scale computational screening of sorbents for adsorption, applicable for a variety of sorbents and gases. As the presented methodology holds strong applicative potential, we provide alongside this paper a modified version of the RASPA2 code with a ghost swap move implementation and a Python library designed to minimize the user’s input for analyzing data derived from the TMMC simulations.

## Introduction

In the coming years, as climate change accelerates, access to potable water may become a luxury. One of the solutions to this pressing issue lies in the extraction of drinking water directly from the atmosphere in a decentralized manner using atmospheric water harvesting (AWH).^1−7^ Materials and even devices that enable this process, have already been demonstrated with metal–organic frameworks (MOFs) emerging as particularly promising sorbent candidates.^8−11^ MOFs offer advantages such as relatively low synthesis costs, the ability to tailor their pores’ shape and size to the desired profile of the water adsorption isotherm, and stability in an aqueous environment, allowing for multiple adsorption–desorption cycles.

Despite the continuous advancement of sophisticated experimental methods to study adsorption phenomena with ever deeper understanding at the microscopic/atomistic scale, numerical simulations offer a level of resolution, understanding, and control over the investigated system that the physical experiment have yet to achieve. Therefore, they remain a remarkably powerful tool for delving into the microscopic world, and for modern material design. This is particularly true when examining MOFs and processes in which they are involved.

Given the modular nature of MOFs, the number of potential structures is theoretically infinite and at least difficult to estimate. The CSD MOF database contains over 12 000 experimentally synthesized MOFs,^12^ CoRE MOF database exceeds 14 000,^13^ and the hMOF database comprises more than 51 000 hypothetical MOFs.^14^ Conducting experimental investigation on such a vast array of materials and the selection of the most suitable one for a specific application is prohibitively costly in terms of time and resources. Therefore, the utilization of large-scale high-throughput numerical screening methods becomes indispensable.

Although water is one of the most common molecules, essential to many chemical, physical, biological, and technological processes, fully comprehending its interaction with surrounding heterogeneous structures remains challenging. A better understanding of the intricate mechanism of water adsorption in MOFs is essential for optimizing the adsorption process in current materials and designing new ones, not only for water harvesting but also for processes where the presence of water influences the adsorption,^15,16^ separation,^17^ or catalysis^18^ of other molecules.

Among various computational techniques, Monte Carlo simulations in a grand canonical ensemble (GCMC) have proven to be particularly valuable for modeling the adsorption of small molecules on surfaces and in porous structures.^19−22^ However, challenges persist in water adsorption simulations, including long convergence times and frequently observed discrepancies between experimental and numerical results.^23−27^ We believe that they are mostly the consequence of two factors: (i) incomplete description of the water interaction with its heterogeneous surroundings, often neglecting strong polarization effects that significantly influence adsorption^28^ (interactions are typically described using rigid, nonpolarizable models); and (ii) high energetic barriers between states, resulting from very strong interactions within water itself, primarily through hydrogen bonding; these barriers often cause the GCMC simulations to become trapped in local energy minima.^29^ In this work, our aim is to address the latter issue; the former requires separate investigation.

To solve the problem of long-living metastable states, instead of standard GCMC algorithm, we employed the grand canonical transition matrix Monte Carlo (GC-TMMC) method using *NVT + ghost swap* approach. Recent studies have shown that this technique significantly enhances the computational efficiency of water adsorption simulations.^29,30^ It consists in conducting simulations in canonical ensemble in which only *ghost* trial insertion or deletion of a molecule (a swap move) is performed but never accepted; only the transition probability is recorded for further analysis. The *NVT + ghost swap* method has been successfully used in various simulations, including adsorption of rigid and flexible molecules under supercritical conditions,^31^ adsorption of small molecules such as methane in rigid^32^ and flexible^33^ frameworks, and adsorption of water in rigid frameworks.^29^

One of the key advantages of GC-TMMC lies in its ability to study the properties of the adsorbed gas, even when the gas is present in more than one phase.^34^ This allows the analysis of metastable phases, which are responsible for the observation of hysteresis loops on the adsorption–desorption isotherms.^35,36^ As hysteresis is a nonequilibrium phenomenon, its accurate determination depends on the duration of experimental observation.^37^ Consequently, the unambiguous experimental determination of hysteresis is challenging, complicating in turn verification of numerical results. However, GC-TMMC simulations allow direct determination of both the limit of stability of the adsorbed phase and pressure of equilibrium phase transition.^34^

The major drawback of the *NVT + ghost swap* method is the necessity to conduct *N* independent simulations, covering the full spectrum of possible macrostates, ranging from an empty to fully saturated framework. Due to substantial porosity and volume available for fluid in MOFs, the required number *N* of *NVT* simulations remains high, often exceeding 1000.

Recently, a modification of the *NVT + ghost swap* procedure, referred to as the C-map method and dedicated to screening materials in adsorption applications, has been developed.^29^ In this approach, at specified pressure and temperature *(pT)* conditions, a TMMC simulation is conducted at predetermined uptake, and the probabilities of molecule insertion and deletion are calculated and accumulated. Consequently, if the system exhibits a higher probability of accepting insertion, it suggests that at the given *pT* conditions the equilibrium loading is higher than the one at which calculations were performed. Similarly, if the probability of particle removal exceeds that of insertion, the equilibrium loading has a lower value. By conducting such simulations for various loading values, one can determine the equilibrium loading range for a specific material under defined *pT* conditions.

A simplified version of this method was recently used^38,39^ to rapidly assess the hydrophobicity or hydrophilicity of MOFs. The simulation was performed only for a single arbitrarily chosen loading value. In such a situation, assuming a perfectly stepped water adsorption isotherm, if the probability of accepting the insertion of a molecule is greater than the probability of its removal, the material under given *pT* conditions is fully filled (hydrophilic); otherwise, it is considered empty (hydrophobic).

In this paper, we propose a methodology with a computational cost comparable to that of the C-map method, yet it also yields the complete water adsorption isotherm. We introduce an effective interpolation scheme wherein to obtain full adsorption isotherm, direct simulations are conducted only for selected macrostates. This approach reduces the total number (and time) of simulations by 2 orders of magnitude while maintaining good agreement with the isotherms calculated from simulations performed for all macrostates. This represents a significant improvement in methodology, especially for applications such as water harvesting processes and other temperature/pressure swing adsorption processes, where the value of interest is working capacity (i.e., the deliverable amount of water or other gas, in a single adsorption–desorption cycle).^3,40^ This value can be determined from the isotherm or isobar of adsorption.

Then, we present, for the first time, an extrapolation of the free energy landscape using these reduced data sets, from which we estimate the adsorption uptake at temperatures that were not simulated directly. The use of the reweighting and extrapolation techniques results in the significant advantage of the GC-TMMC method over the GCMC method—the ability to determine the adsorption properties of a sorbent (in this case, three selected MOFs) across a continuous range of temperatures and pressures based on calculations conducted *at only one* temperature and pressure. This methodology represents a pioneering approach that enables simultaneously fast, accurate, and efficient high-throughput screening of MOFs for water adsorption applications.

## Methods

Here, we outline the fundamental assumptions of the grand canonical transition matrix Monte Carlo (GC-TMMC) procedure. For a detailed thermodynamic description of the method, we direct the reader to references.^34,41,42^

The main objective of the GC-TMMC method is to calculate the macrostate probability distribution (MPD), instead of direct calculation of the ensemble averages. To achieve this goal, the simulation is biased to sample all macrostates with equal probability. In this way, macrostates with relatively high free energy may be explored. When investigating adsorption phenomena, it is convenient to use the number of particles in the system, denoted as *N*, as a macrostate variable. The MPD in the grand canonical ensemble is then given by
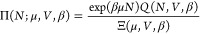
1where μ is chemical potential, *V* is system volume, β = 1/*k*_B_*T* (*T* being temperature and *k*_B_ - Boltzmann constant), and *Q*(*N,V,β*) and Ξ(μ,*V,*β) are respectively canonical and grand canonical partition functions. To determine MPD, each time the system visits a macrostate *N*, the so-called collection matrix (C-matrix) is updated with the unbiased probabilities of accepting a swap move (*p*_acc_), according to
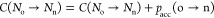
2
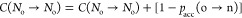
3where the labels o and n refers to *old* and *new* configurations for attempted move. Then, the probability transition matrix is computed by normalization of the C-matrix:
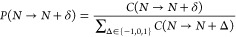
4

The Π(*N*; μ,*V,*β) can be then calculated using relation:
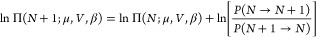
5Using the following protocol: first, an arbitrary value is given to ln Π(*N*_min_), then values of ln Π for next *N* are calculated sequentially. The minimum and maximum particle numbers are set to ensure the sampling of the entire domain of interest, from empty (*N*_min_ = 0) to fully saturated system (with *N*_max_ calculated from the volume of the system available for adsorption; for details read section Simulation Details in Supporting Information). To ensure that the entire domain of interest has been sufficiently sampled, one should always check whether Π has a sufficiently low value in *N*_max_:
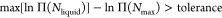
6where *N*_liquid_ corresponds to the macrostates in the liquid domain. In our simulations, we used the tolerance of 10 as the states of relative probability of *e*^–10^ do not contribute significantly to the ensemble averages.

To calculate the ensemble average of any physical quantity *A* in the grand canonical ensemble, we use the equation:^43^
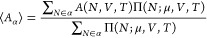
7where the sums are calculated over the macrostates that belong to phase α and *A* denotes value of interest which is accumulated for each visited macrostate *N*. The GC-TMMC simulations performed at one μ value can be easily recalculated for any other μ′ value by simple histogram reweighting using relation:^42^
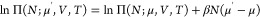
8

In this way, from a single set of calculations (performed at single *μ*) we can obtain the full isotherm at infinite resolution. For a more detailed description of the identification of phases and the calculation of thermophysical properties from MPD, we refer the reader to the paper by Siderius et al.^34^

To compute MPD, it is essential to sample all relevant macrostates to gather adequate transition statistics. Typically, this is achieved in a multiple-macrostate approach, where a single simulation sweeps through a range of adsorbed particles. To sample low-probability states, a biasing function is employed, such as in the Wang–Landau (WL) algorithm.^44,45^ It is also possible to use a single-macrostate approach, where *N* simulations in NVT ensemble are conducted, which artificially imposes equal sampling of a domain and allows for the utilization of the TMMC principles without employing a biasing function.^29,31,33^ In this case, transition probabilities are calculated using the so-called *ghost swap* move. This move resembles a standard swap move, involving a trial insertion or deletion of a molecule, but it is never accepted: only transition probabilities are recorded. Consequently, the total number of molecules remains constant through simulation. Hatch et al.^46^ demonstrated that single-macrostate simulations are less efficient than multiple-macrostate simulations, mainly due to the lack of microstate sampling. However, we show that with single-macrostate simulations, it is possible to skip sampling certain macrostates and interpolate transition probabilities to obtain the full MPD with reduced simulation cost. At the same time, in this study, we do not undertake a comprehensive analysis of the differences in efficiency among various approaches, which we discuss in detail in the last part of the Results and Discussion section.

**Figure 1:**
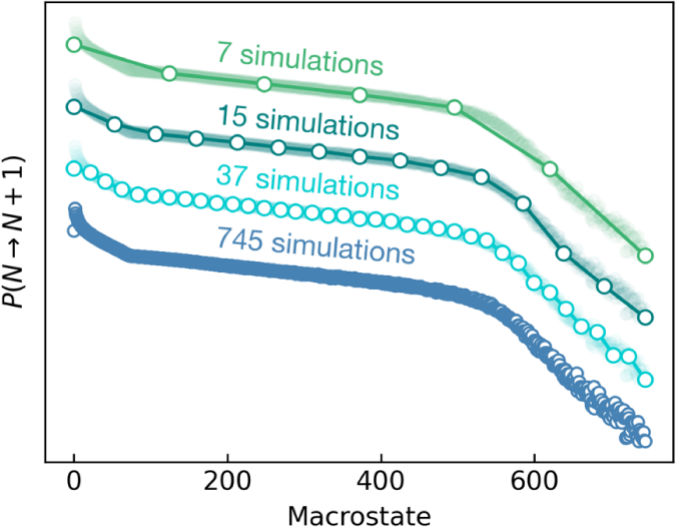
Macrostate representation for MOF-303. Macrostates for which the transition probabilities were explicitly calculated are represented by open symbols; macrostates with interpolated transition probabilities are marked with a dark line connecting open points. The light bold lines indicating the reference macrostates are provided for better comparison of the used macrostates reduction.

In this work, *NVT + ghost swap* simulations were conducted for each *N* within the macrostate range using modified version of RASPA2 code.^47^ In the Supporting Information, we also compare the results obtained from these simulations with those obtained using the WL/TMMC method to eliminate the risk of referring to erroneous results. Following the simulation, we reduced the *NVT + ghost swap* simulation data sets by factors of 20, 50, and 100. Table 1 provides the exact numbers of macrostates in the reduced data sets for each system studied: MOF-303,^48^ MOF-LA2–1,^9^ and NU-1000.^49^ Transition probability values for macrostates not included in the reduced data sets were calculated by linear interpolation between the nearest states in the simulation. A visualization of such an approach for MOF-303 is presented in Figure 1. The selection of these materials was based on their spectrum of isotherms, which closely approximates the typical isotherms of water adsorption in high-potential MOFs for use in AWH.^40^ This encompasses adsorption by continuous pore filling (MOF-303), adsorption with stepped isotherms with minimal presence of metastable states (MOF-LA2–1), and adsorption with stepped isotherms with pronounced and long metastable states (NU-1000). The rationale behind the choice of materials is discussed in detail in the Choice of Porous Materials section in Supporting Information.

**Table 1:** Number of Macrostates for which Direct Transition Probability Calculations Were Performed

reduction factor	MOF-303	MOF-LA2–1	NU-1000
reference	745	889	1200
20	37	44	60
50	15	18	24
100	7	9	12

To extrapolate the free energy landscape obtained at single simulation temperature to other temperatures, we followed the method published by Mahynski et al.^50,51^ The ln Π(N) is expanded in a Taylor series:
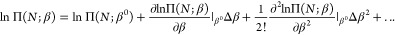
9where Δβ = β – β_0_ and β_0_ is the inverse simulation temperature. In the grand canonical ensemble, the first two terms of the Taylor expansion simplify to
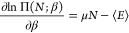
10
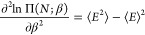
11where *E* is the potential energy and ⟨*E*⟩ is the ensemble average potential energy in the canonical ensemble. In this study, all extrapolated isotherms are calculated using only the first order Taylor expansion, since the next terms converged much more slowly. In most cases, it caused significant noise, which considerably affected the obtained results, as discussed in detail in the Supporting Information.

## Results and Discussion

### Definition of the Problem and Proposed Methodology

**Figure 2:**
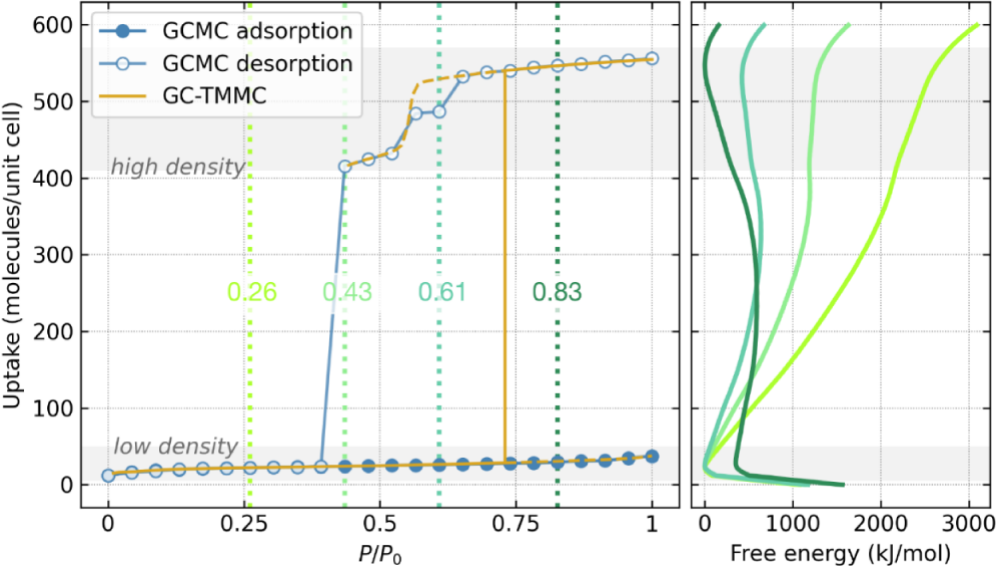
(left) Isotherms of water adsorption in NU-1000 at 298 K simulated using the GCMC (2 × 10^6^ and 1 × 10^6^ cycles for adsorption and desorption, respectively) and GC-TMMC (10^5^ cycles) methods. The solid yellow line represents a track of stable states; the dashed yellow line represents metastable branches of the isotherm. GCMC adsorption simulation was started with an empty system. GCMC desorption simulation was started with system containing 528 molecules/unit cell that was already equilibrated in NVT simulation (for water cluster formation). In GCMC simulations, during both adsorption and desorption, insertion and removal of molecules were allowed. (right) Free energy profiles at relative pressure indicated by a vertical line of the corresponding color on the plot on the left.

We begin by justifying the choice of the grand canonical transition matrix Monte Carlo (GC-TMMC) method for simulations of water adsorption. Our case study is water adsorption in NU-1000 at temperature *T* = 298 K. First, we conducted two series of calculations using the GCMC method: (i) starting from an empty system, we calculated the adsorption isotherm, and (ii) starting with a pre-equilibrated system containing 528 molecules/unit cell (corresponding to a fully saturated system), we calculated the desorption isotherm (Figure 2, left panel). Both curves served as reference for subsequent analysis. Due to the slow convergence of GCMC water adsorption simulations, extensive calculations were conducted, comprising 2 × 10^6^ cycles for adsorption simulations and at least 1 × 10^6^ cycles for desorption, with the longest desorption simulation spanning 90 days. Despite the considerable duration of these simulations, the system has not reached the equilibrium state. In contrast, using the GC-TMMC method, we conducted only one set of calculations at *T* = 298 K and *p* = 3200 Pa, comprising only 10^5^ cycles in production runs. From these calculations, using reweighting, we were able to determine both the low- and high-density branches of the isotherm (referred to as gas and liquid branches, respectively), phase stability ranges, and equilibriumtransition pressure. Additionally, from the same simulation, we calculated the free energy of a system at various pressures (Figure 2, right panel). This allowed us to explain both the mechanism of water adsorption in NU-1000 and why GCMC simulations did not deliver an equilibrium isotherm.

As presented in Figure 2 (left), the GC-TMMC adsorption isotherm aligns with the GCMC gas phase branch across the entire pressure range, even as the latter becomes metastable. Similarly, the liquid phase branch of the GC-TMMC desorption isotherm coincides with the GCMC throughout the pressure range where the liquid phase occurs. As the metastable liquid phase vanishes, the system is emptying and the desorption branch merges with the gas phase branch, the only phase that exists at low pressure.

In principle, the GCMC isotherms should follow only stable states in both adsorption and desorption simulations. However, this is not the case because simulations often become trapped in local energy minima. To illustrate this phenomenon, we calculated (from GC-TMMC simulations) the free energy profiles at various pressures: (i) when only the gas phase is present, (ii) when the liquid phase is metastable, and (iii) when the gas phase is metastable (see Figure 2, right panel). We observe the emergence of two minima—one at low density (*ld*) and the other at high density (*hd*), corresponding to uptakes of ∼30 and ∼500 molecules per unit cell, respectively. Even at saturation pressure, starting the GCMC simulation from an empty system does not lead to the increase of the adsorbed amount. In fact, despite a free energy difference of approximately 400 kJ/mol favoring the *hd* state, the energy barrier (∼200 kJ/mol) between the *ld* and *hd* states is roughly 80 times higher than the thermal energy *k*_B_*T* at 298 K, causing the system to remain blocked in the local minimum in the *ld* state. Similarly, when the simulation begins with a fully saturated system, at *P*/*P*_0_ = 0.43, it remains trapped at the local minimum in the *hd* state, despite a free energy difference of about 1200 kJ/mol in favor of the *ld* state. Again, the barrier between the *ld* and *hd* states exceeds the thermal energy. As the pressure decreases, the local minimum associated with the *hd* state disappears, and the simulation initiated from the *hd* state at *P*/*P*_0_ = 0.26 easily reaches the equilibrium state at *ld* as there is no energy barrier hindering the process.

Consequently, we constate that, compared to standard GCMC simulations, the GC-TMMC method offers increased accuracy, reduced simulation time, and deeper understanding of the thermodynamic information of the system under investigation.

### Isotherms from Reduced Data Sets

As previously mentioned, the main limitation of the *NVT + ghost swap* method is the number of simulations needed to calculate the macrostate probability distribution (MPD), which serves as the basis for extracting all thermodynamic properties of a system. We show that it is possible to conduct simulations at reduced number of macrostates and subsequently interpolate the missing ones from the directly calculated values.

**Figure 3:**
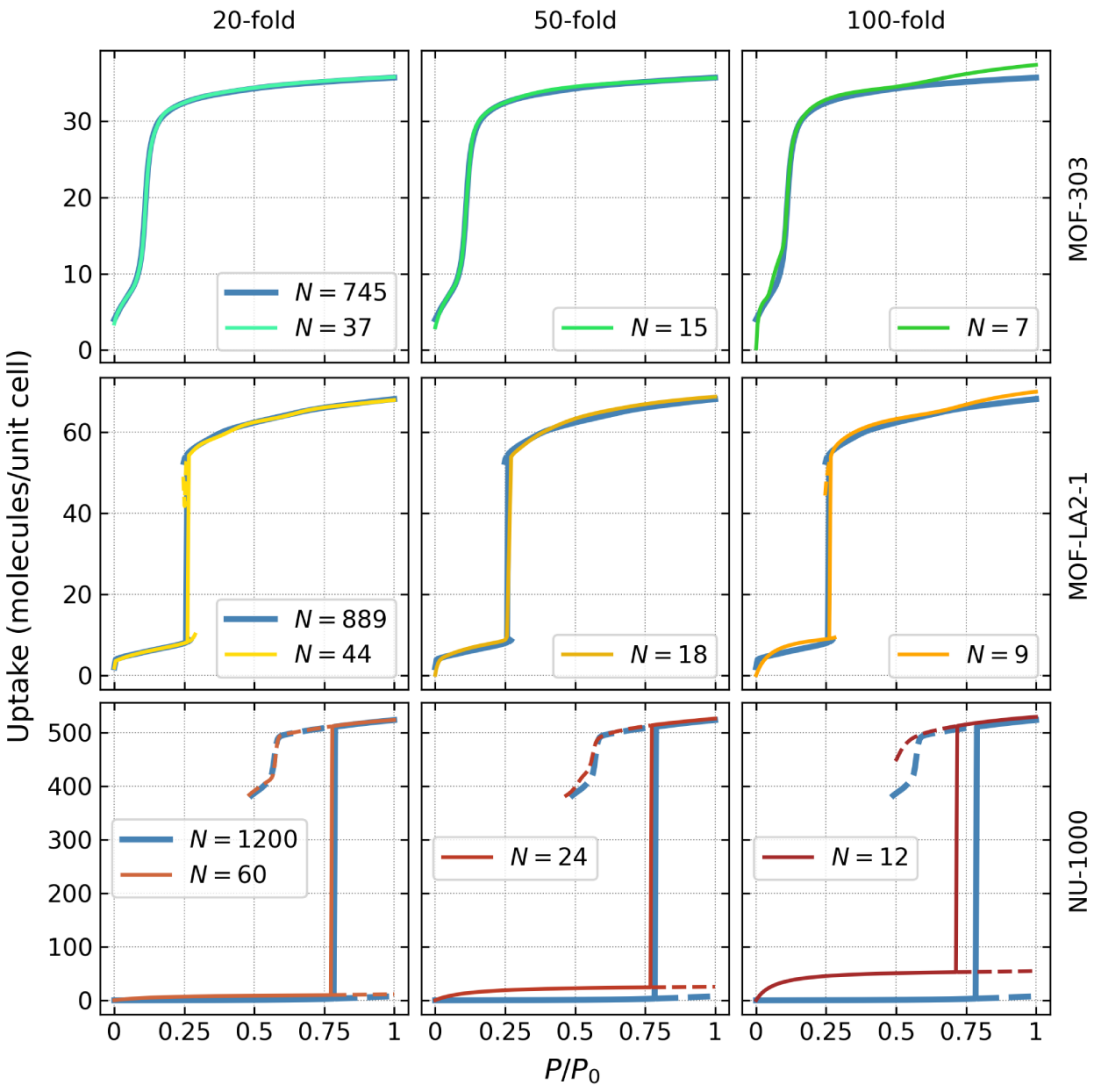
Isotherms of water adsorption at 298 K calculated for three MOFs: (top) MOF-303, (middle) MOF-LA2–1-ZUS, and (bottom) NU-1000, using data sets reduced (left) 20 times, (middle) 50 times, and (right) 100 times. The reference isotherms (calculated using full data sets) are colored with a slightly wider blue line for better visibility.

To assess the accuracy of interpolation of transition probabilities, we first conducted simulations across all macrostates for three systems: MOF-303, MOF-LA2–1, and NU-1000. Then, during postprocessing, we reduced the number of macrostates for which transition probabilities are known directly 20, 50, and 100 times (Table 1). The resulting isotherms are presented in Figure 3. For all three MOFs, the isotherms calculated using data sets reduced 20 and 50 times show good or near-perfect agreement with the reference isotherms. Upon further reduction in the number of simulations (by 100 times), we observe larger (albeit still acceptable) discrepancies: (i) for MOF-303, the uptake is slightly underestimated at lowest pressure and overestimated at highest pressure; (ii) for MOF-LA2–1 the saturation amount and location of the step on isotherm are accurately predicted, with minor differences in gas branch; however, these differences are not significant enough to impact the analysis of the results; (iii) for NU-1000, differences are more pronounced—while the saturation volume is reproduced correctly, the uptake in the gas phase and the step pressure slightly deviates from the reference values.

We observe a distinct trend in which, as the system size expands, and consequently the energy barrier between states increases, the reduction in direct simulations results in a noticeable degradation of the calculated adsorption isotherms. One factor contributing to this result may be that as the size of the system increases, the range of macrostates that separate the phases broadens. Consequently, more simulations fall within the range of low probability states compared to the number of simulations that sample high probability states.

However, when searching for optimal materials for atmospheric water harvesting applications, we prioritize specific characteristics, including (i) the relative humidity (or P/P_0_) at which the material adsorbs water, (ii) shape of the isotherm (a step-shaped isotherm being most desirable), (iii) the volume of water that can be collected in a single adsorption–desorption cycle, (iv) the hydrothermal stability of the material (to ensure long-term operation), and (v) components used for adsorbent synthesis (preferably, nontoxic, and abundant). The first three features can be confidently obtained directly from short *NVT + ghost swap* simulations. According to Figure 3, even with data sets reduced 100 times (thus calculated using only 7, 9, and 12 macrostates for MOF-303, MOF-LA2–1, and NU-1000, respectively), these characteristics are in perfect or good agreement with reference values. In the Supporting Information, we present the calculation of working capacity for NU-1000, identified as the worst performing material in this study. We show that the error for the calculated values is only 1.16%, 3.60%, and 8.51% for simulations performed with 60, 24, and 12 macrostates, respectively (Table S2).

A direct comparison of the computational times of GCMC and GC-TMMC is not straightforward, given their different approaches of phase space sampling. In GCMC simulations, a single simulation provides an average uptake under specific *pT* conditions. In contrast, in GC-TMMC, a series of simulations for different uptake values are conducted, from which an isotherm with infinite resolution is derived through pressure reweighting in postprocessing. Consequently, the time required for a single simulation in GC-TMMC depends on the number of molecules present in the system (thus size of the system), rather than the selected pressure. For this reason, for some hydrophilic MOFs (such as MOF-303 or MOF-LA2–1) that do not present a challenge to GCMC simulations, the computational cost required to calculate a few isotherm points might be similar to performing a series of GC-TMMC simulations, assuming the interpolation scheme proposed in this work is used. However, predicting the simulation outcome prior to its execution is not possible. Furthermore, it is only after completing a GCMC simulation that one can determine whether the simulation will be challenging or not. Additionally, if the GCMC simulation becomes trapped at a local energy minimum, fluctuations in properties as a function of the number of MC cycles may falsely indicate equilibrium (Figure S7). In conclusion, both methods can achieve similar performance for the most hydrophilic MOFs; however, GC-TMMC offers better control over simulation convergence. Furthermore, other benefits of the GC-TMMC method, such as infinite isotherm resolution, access to the system’s free energy, ability to explore metastable states, and temperature extrapolation, should also be taken into account.

### Temperature Extrapolation

One of the major advantages of the TMMC method is its capability to extrapolate the free energy landscape computed at a single temperature to other temperatures through simple postprocessing of the data.^50,51^ Here, for the first time, we evaluate the accuracy of this extrapolation technique while simultaneously reducing the number of simulations. We limited the analyzed temperatures to the 298 K–343 K range, as this represents an operating range for water adsorption in MOFs.

**Figure 4:**
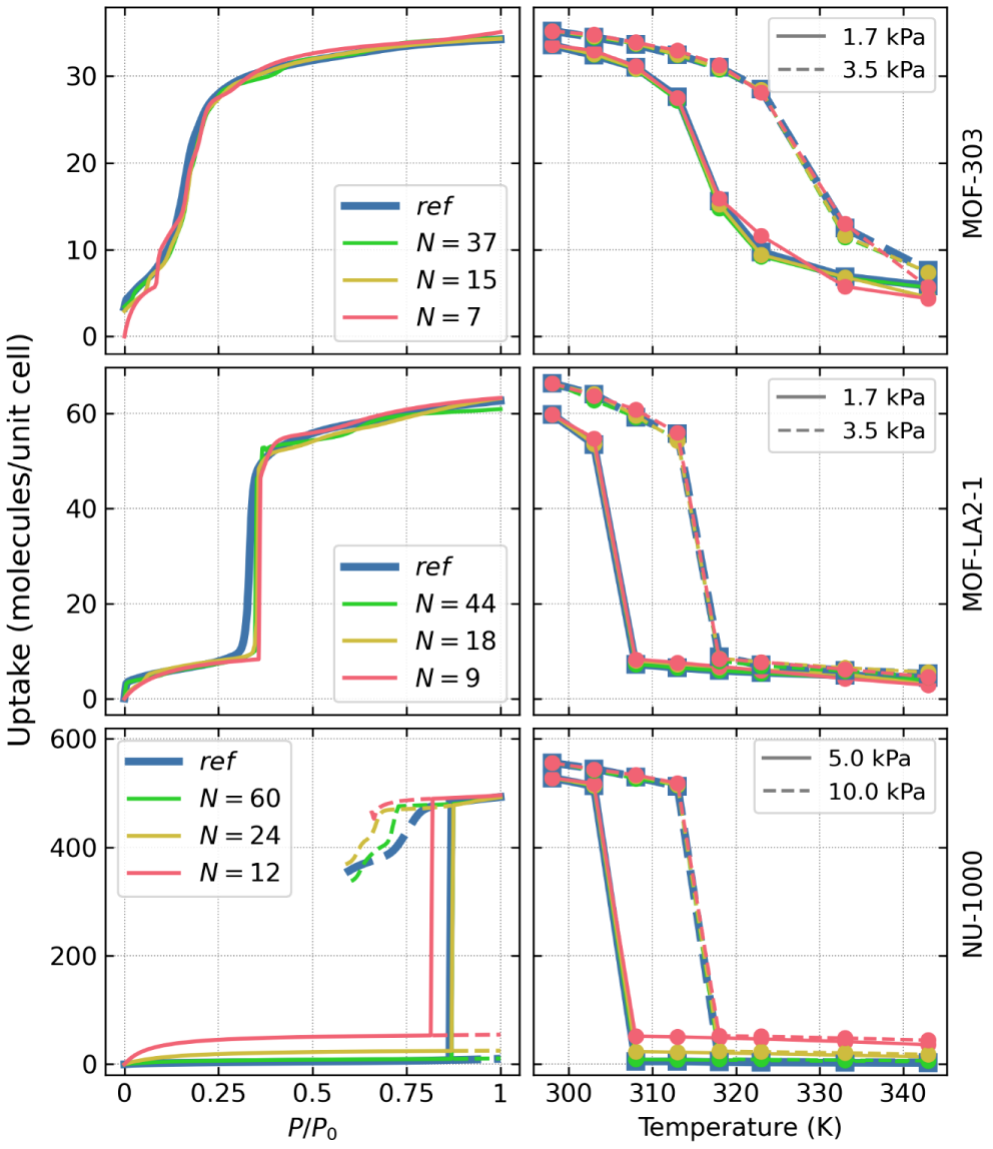
Extrapolated isotherms (*T* = 343 K, left) and isobars (at 1.7 and 3.5 kPa for MOF-303 and MOF-LA2–1, and at 5.0 and 10.0 kPa for NU-1000, right). The reference isotherms (in blue) were calculated for each MOF in direct simulations at *T* = 343 K. All other curves were extrapolated from simulations performed at *T* = 298 K using the number of states indicated in the legend.

Figure 4 shows isotherms and isobars calculated using the *NVT + ghost swap* method and the extrapolation technique. The left panel presents water adsorption isotherms at 343 K, extrapolated from data collected at 298 K with a reduced number of macrostates. In all cases, the extrapolated isotherms agree well or nearly align with the reference isotherms computed directly at 343 K across the entire pressure range. When the number of macrostates decreases, some minor differences appear in the low-density range and in the metastable branches; however, the most important characteristics of the isotherm (such as the pressure of the *ld* to *hd* transition or total uptake) remain in good agreement with simulation performed directly at 343 K. Considering the results obtained for 298 K with interpolation of transition probabilities (as shown in Figure 3), a similar degree of decrease in accuracy is observed during simultaneous extrapolation and interpolation. Consequently, within the studied range of temperatures, the observed error is mainly due to interpolation of the results rather than their extrapolation.

This observation represents a substantial advancement in the screening capabilities of simulations. So far, most screening procedures employing classical simulation methods have focused on extracting a single material characteristic (in most cases, the maximal loading at a selected pressure). With the *NVT + ghost swap* method, we can derive thermodynamic data for a diverse range of temperatures and pressures from just a few simulations with practically infinite resolution, given that extrapolation and pressure reweighting are obtained during data postprocessing.

Most industrial applications of adsorption phenomena use temperature/pressure swing adsorption (T/PSA) procedures, where cyclic changes in pressure and/or temperature prompt alternating adsorption and desorption of fluid. The working capacity of a particular sorbent is evaluated as the amount of fluid that can be recovered (or harvested, in the case of water adsorption from the atmosphere) in one adsorption–desorption cycle. This quantity can be easily calculated from the adsorption isotherms/isobars at various temperatures/pressures of interest as a difference in the average uptake between any two (*p*_1_*T*_1_), (*p*_2_*T*_2_) state points.

To illustrate this approach, the right panel of Figure 4 shows the adsorption isobars for the discussed systems. Let us consider determining the working capacity of the MOF for water adsorption at *T* = 298 K and *p* = 3.5 kPa and the desorption at *T* = 333 K and *p* = 1.7 kPa. By comparing MOF-303 and MOF-LA2–1, we can conclude that under identical operating conditions MOF-LA2–1 yields approximately twice as many molecules of water per unit cell as MOF-303 (60 molecules/unit cell vs 30 molecules/unit cell, respectively).

### *NVT + Ghost Swap* Convergence Time

**Figure 5:**
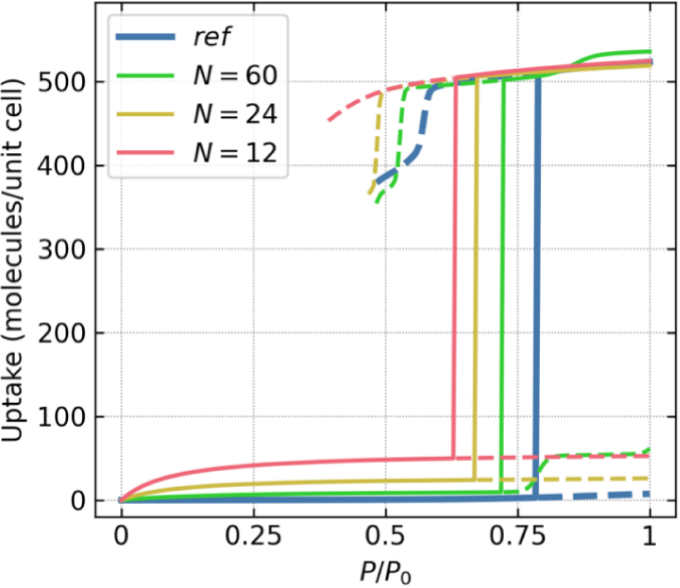
Isotherm of water adsorption in NU-1000 at 298 K calculated with 5 × 10^3^ production cycles and variable number N of macrostates used in the calculation of direct transition probabilities. Solid and dashed lines correspond to stable and metastable states, respectively.

The main constrain in water adsorption screening studies using classical simulation methods is the extremely long simulation time. This issue is solved within the *NVT + ghost swap* method. Therefore, we also verified (following previously discussed interpolation technique) how the number of simulation cycles affects the isotherm profile. The results are presented in Figure 5: isotherms derived from data collected after 5 × 10^3^ cycles of simulations closely reproduce the reference isotherm, even using the minimal number of simulations. As presented in Figure 2, the isotherm derived from the GCMC simulations failed to converge to equilibrium values despite conducting simulation with a number of cycles 3 orders of magnitude higher than those presented in Figure 5. This suggests that the presented approach (short, low-demand simulations) can serve for preliminary materials selection in large-scale screening studies, allowing more accurate (longer) calculations to be performed on already narrowed selection of sorbents.

The efficiency of the presented *NVT + ghost swap* method compared to the multiple-macrostate TMMC method remains an open question, particularly when considering the advantages of probability interpolation. Results presented by Hatch et al.^46^ suggest that for adsorption in a repulsive porous network, single-macrostate simulations might be 3 orders of magnitude less efficient than double-macrostate simulations, due to the lack of microstate sampling. However, their study focused on gas adsorption in a purely repulsive porous network, potentially corresponding to a homogeneous hydrophobic material. In contrast, MOFs are purely heterogeneous materials with diverse interactions with adsorbing molecules, from strongly repulsive to strongly attractive. Moreover, the authors agreed that the choice of sets of Monte Carlo moves significantly impacts the difference in performance between single- and double-macrostate simulations. Furthermore, the object of comparison was the natural logarithm of macrostate probability distribution, which converges relatively slowly, while the resulting average number of adsorbed molecules reaches the equilibrium value more rapidly. This is a consequence of the fact that the MPD quickly reaches values close to equilibrium values, while exhibiting significant noise that slowly disappears with increasing number of MC moves (Figure 7 in cited paper^46^). This noise, however, has little effect on the average number of molecules, as the average is strongly dependent on the location of the peak on the MPD profile because values from this region have largest weight, which follows directly from eq 7.)

## Conclusions

In this study, we presented and explored the practical application of the TMMC method in the *NVT + ghost swap* approach for computation of water adsorption isotherms in MOFs. This method uses the interpolation of transition probabilities to reduce computational cost while maintaining the accuracy of the results. Importantly, the TMMC method provides considerably more thermodynamic insight into adsorbing systems compared to GCMC simulations. In the case of water adsorption, it significantly reduces the calculation time and improves the precision due to overcoming the issues associated with simulation getting trapped in local energy minima. Moreover, for the first time, we combined interpolation of probabilities with temperature extrapolation method, obtaining high-quality results at temperatures beyond those directly simulated.

In the context of water harvesting, our study demonstrated that the working capacity of the sorbent, crucial for T/PSA processes, can be calculated with very high accuracy across an extensive range of pressures and temperatures using exclusively a single set of simulations conducted at single temperature and pressure. This allows for a rapid and effective material preselection for a given application and facilitate the optimization of the adsorption process itself. In particular, this methodology marks a significant step toward the advancement of large-scale computational screening of MOFs for water (and other gases) adsorption applications. Moreover, MPD data can be stored and easily recalculated for different thermodynamic conditions, aligning the described methodology with the current trend of working with reusable data.^52^ Furthermore, since MPD and free energy are directly related, these data can potentially be used for training machine learning algorithm for a more efficient search for new materials.^53,54^

To enhance the accessibility of the presented methodology, which holds strong applicative potential, we provide alongside this paper a modified version of the RASPA2 code^47^ with a *ghost swap* move implementation. Additionally, we provide a Python library designed to minimize the user’s input for analyzing data derived from the TMMC simulations. This library simplifies the interpolation of probabilities and extrapolation of MPD and allows for automatic calculation of isotherms (including writing to the AIF file^55^ for data standardization purposes).

Future research could be dedicated to better understanding of the influence of all factors affecting the course of TMMC simulations of gas adsorption in MOFs. Furthermore, given the low acceptance rate of MC moves when simulating water adsorption and the formation of highly ordered water network during adsorption, to further accelerate water adsorption simulations advanced MC moves must be explored.

## Data Availability

Modified RASPA2 code for GC-TMMC simulations, python code for TMMC data analysis with example Jupyter Notebook and all exemplary simulation input files are available at https://github.com/b-mazur.

## Supporting Information Available

The following files are available free of charge. Details on simulation methodology used in this work, additional simulation results of adsorption isotherms, and tables and figures referred in the main text (PDF)
