# Supplementary material for: Efficient Modeling of Water Adsorption in MOFs Using Interpolated Transition Matrix Monte Carlo
Source: ACS Appl Mater Interfaces. 2024 May 6;16(19):25559–67. doi: 10.1021/acsami.4c02616 (PMC11103664; doi:10.1021/acsami.4c02616)
Supplement: Supplementary file 1 — am4c02616_si_001.pdf [file am4c02616_si_001.pdf]

Supporting Information

Efficient Modelling of Water Adsorption in MOFs Using Interpolated Transition Matrix Monte Carlo

Bartosz Mazur[1], Lucyna Firlej[1,2], Bogdan Kuchta[1,3]**

[1] Department of Micro, Nano, and Bioprocess Engineering, Faculty of Chemistry, Wroclaw University of Science and Technology, 50-370 Wroclaw, Poland
[2] Laboratoire Charles Coulomb (L2C), Universite de Montpellier - CNRS, 34095 Montpellier, France
[3] MADIREL, CNRS, Aix-Marseille University, 13013 Marseille, France

Bartosz Mazur: bartosz.mazur@pwr.edu.pl
Bogdan Kuchta: bogdan.kuchta@pwr.edu.pl

TABLE OF CONTENTS

USE OF THE HIGHER ORDER TAYLOR TERMS....3
CHOICE OF POROUS MATERIALS....8
SIMULATION DETAILS....10
WL/TMMC VALIDATION....11
WORKING CAPACITY CALCULATION....12
SIMULATION CONVERGENCE....13
COMPARISON WITH EXPERIMENTAL DATA....15
FREE ENERGY MAP....17
REFERENCES....18

USE OF THE HIGHER ORDER TAYLOR TERMS

As described in the main text to extrapolate the free energy landscape to temperatures different from the simulation temperature, the $\ln \Pi(N)$ is expanded in a Taylor series (Equation 9 in main text). Here we tested how the isotherm changes depending on the number of terms used. Figure S1 presents isotherms of water adsorption in MOF-303 extrapolated from transition probabilities collected with different number of simulation cycles and extrapolated using only first or up to second Taylor expansion term. For better understanding in Figure S2 we presented the absolute error calculated between the reference isotherms (calculated directly at given temperature) and the extrapolated isotherm. A similar analysis for MOF-LA2-1 is presented in Figures S3 and S4. An important observation is that in all cases the extrapolated isotherms are in good or perfect agreement with the reference one. However, with lower number of production cycles use of the second Taylor term introduces some noise in calculated isotherms, which increases with increasing difference between temperature of simulation and extrapolation. As the number of simulation cycles increases, this noise disappears. This clearly comes from the fact that the higher order terms include fluctuations of the energy, and these converge much slower than just the mean potential energy of the system. Considering that the next order terms of the Taylor series do not significantly improve the extrapolated isotherm, and possibly introduce some noise, we decided to use only the first term in our calculations.

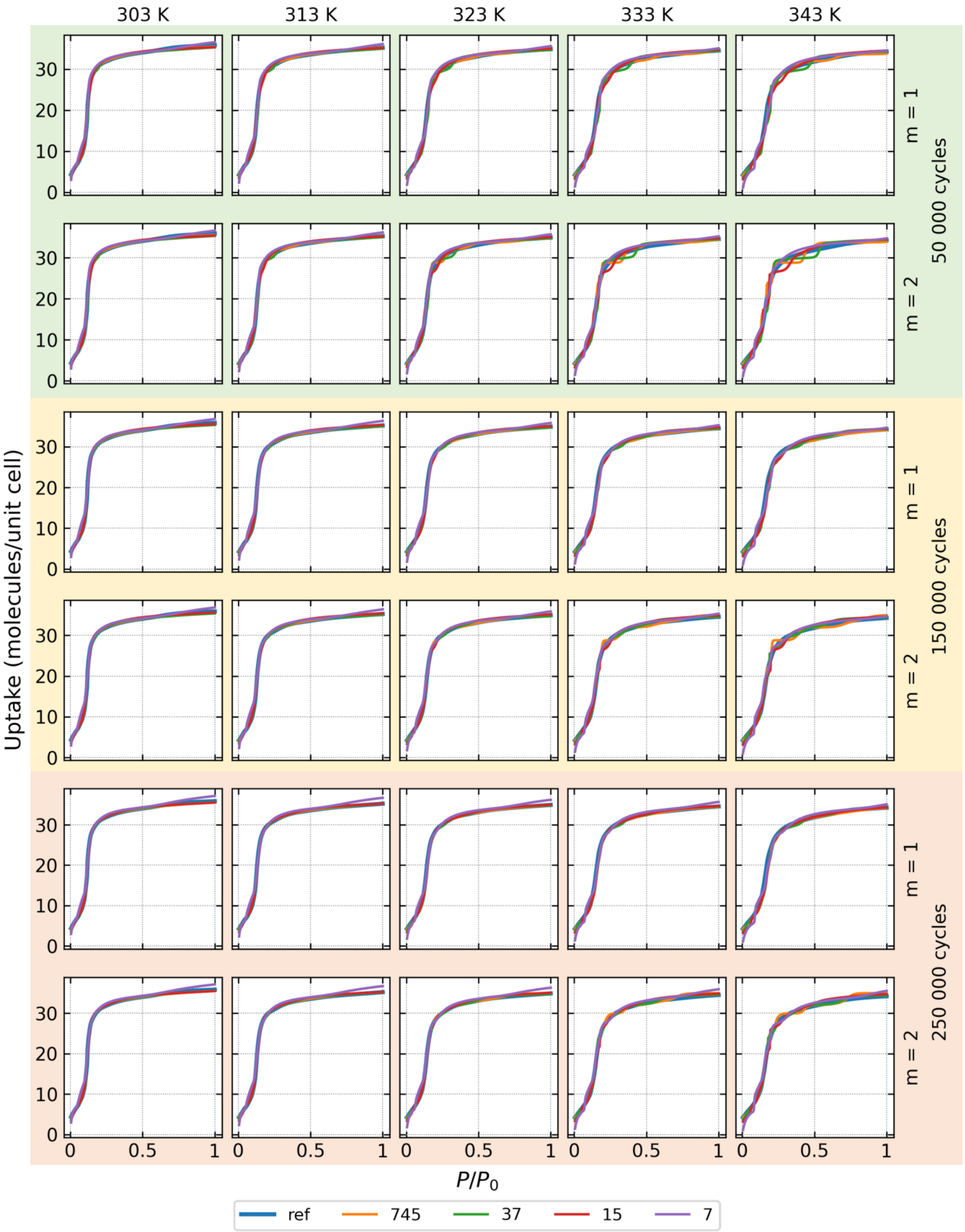

Figure S1. Isotherms of water adsorption in MOF-303 extrapolated from MPD collected at 298 K with 50 000, 150 000, and 250 000 production cycles (green, yellow, and red background, respectively). The value of m indicates how many Taylor series terms were used for extrapolation. The thick blue isotherm is a reference isotherm calculated from MPD collected directly at given temperature, and the orange, red, green, and violet corresponds to data extrapolated from 298 K using 745, 37, 15, and 7 direct simulations of transition probabilities.

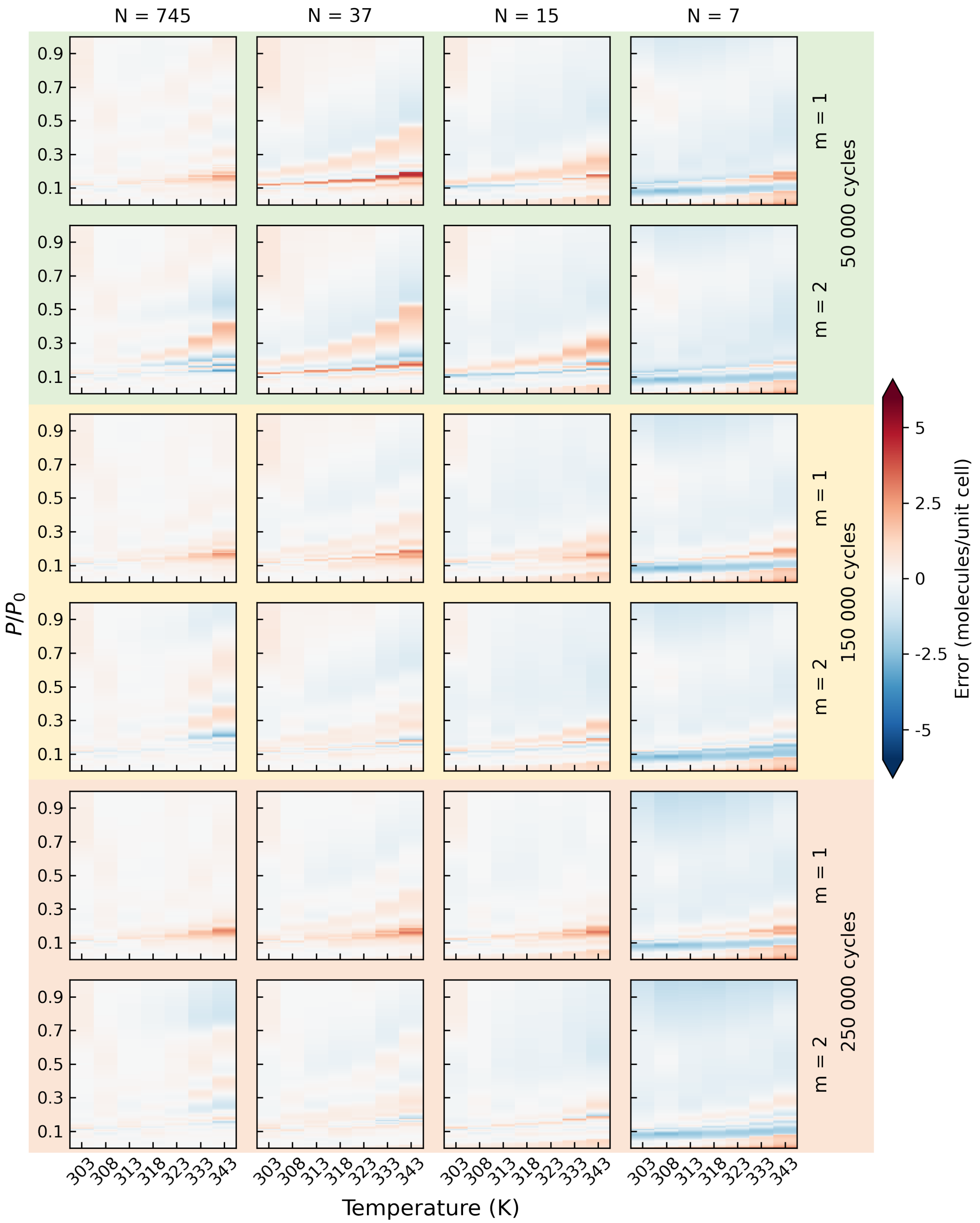

Figure S2. Relative errors for isotherms of water adsorption in MOF-303 extrapolated from 298 K. Each column represents the number of macrostates at which transition probabilities were collected directly. Background color indicates the number of cycles used for isotherm calculation (green, yellow, and red for 50 000, 150 000, and 250 000 cycles, respectively). The value of m indicates how many Taylor series terms were used for extrapolation.

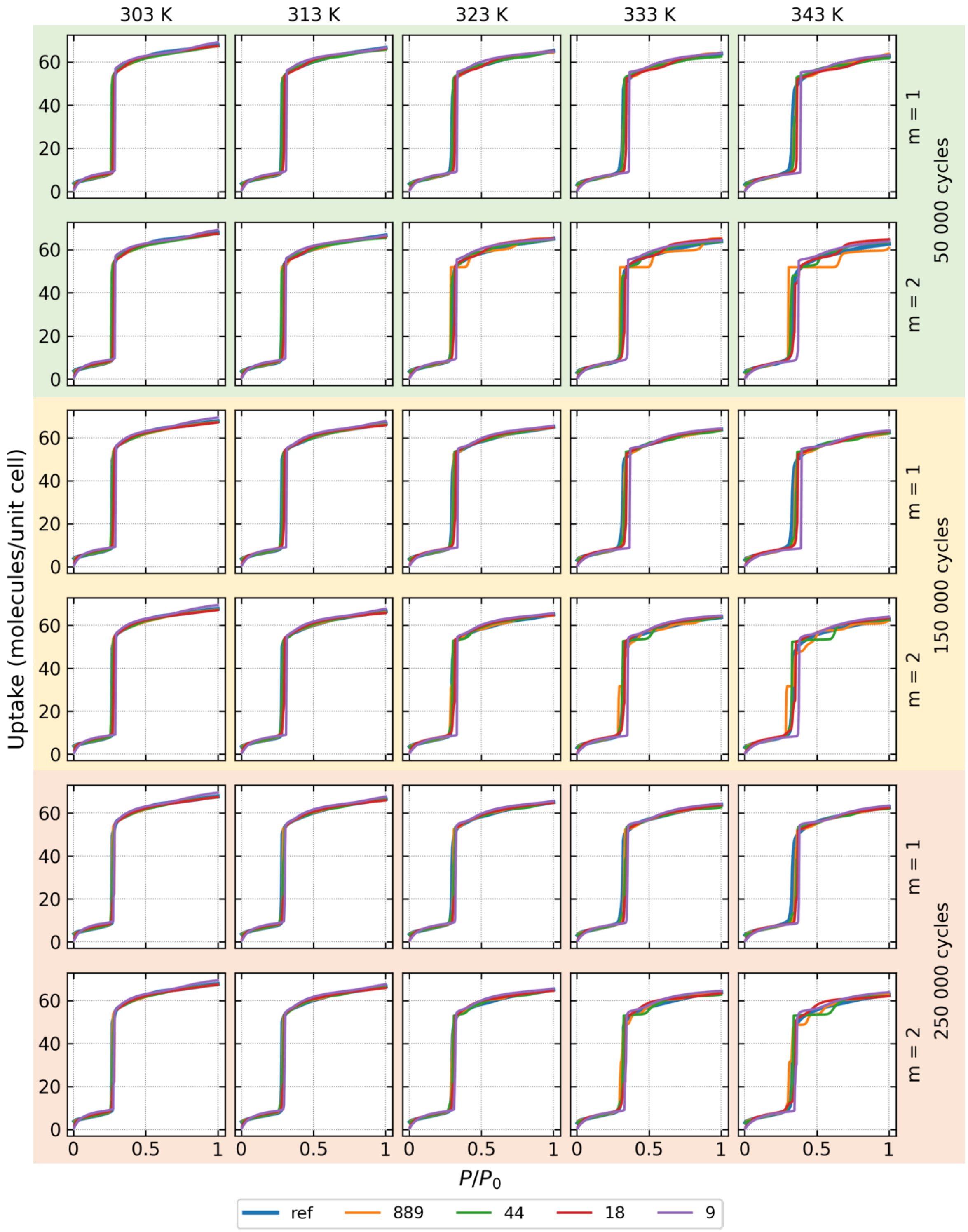

Figure S3. Isotherms of water adsorption in MOF-LA2-1 extrapolated from MPD collected at 298 K with 50 000, 150 000, and 250 000 production cycles (green, yellow, and red background, respectively). The value of m indicates how many Taylor series terms were used for extrapolation. The thick blue isotherm is a reference isotherm calculated from MPD collected directly at given temperature, and the orange, red, green, and violet corresponds to data extrapolated from 298 K using 889, 44, 18, and 9 direct simulations of transition probabilities.

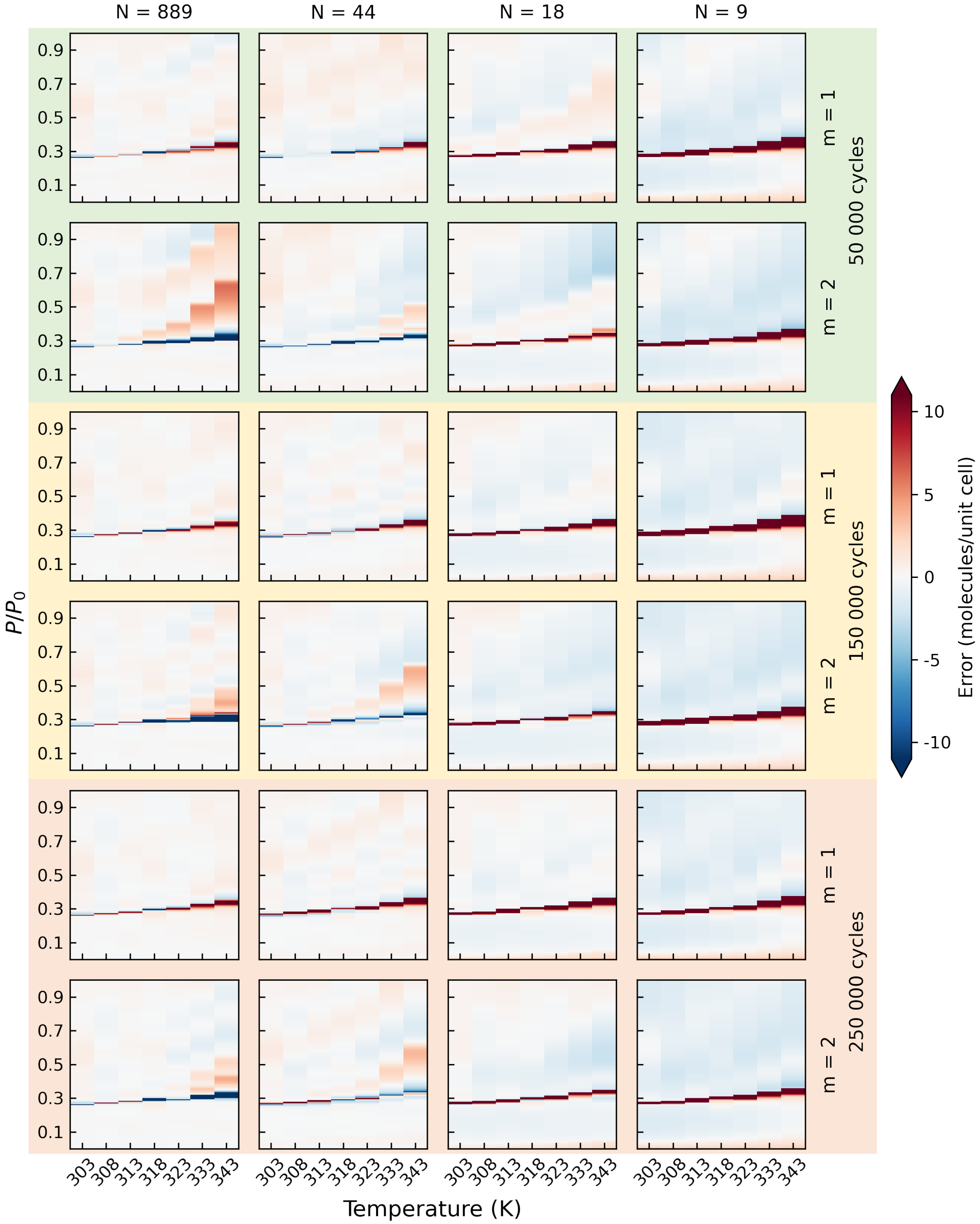

Figure S4. Relative errors for isotherms of water adsorption in MOF-LA2-1 extrapolated from 298 K. Each column represents the number of macrostates at which transition probabilities were collected directly. Background color indicates the number of cycles used for isotherm calculation (green, yellow, and red for 50 000, 150 000, and 250 000 cycles, respectively). The value of m indicates how many Taylor series terms were used for extrapolation.

CHOICE OF POROUS MATERIALS

In this work, we focus on the adsorption of water on three different MOFs – MOF-303[1], MOF-LA2-1[2], and NU-1000[3]. We carefully selected these MOFs because they reproduce a spectrum of typical water adsorption mechanisms that occur in MOFs[4]. MOF-303 and MOF-LA2-1 are aluminium oxide rod-based metal-organic frameworks that are state-of-the-art adsorbents for atmospheric water harvesting applications. MOF-LA2-1 was developed using a linker extension strategy that involves adding a single vinyl group to the $PZDC^{2-}$ linker used in MOF-303, which increases the pore volume from 0.48 cm^3/g to 0.67 cm^3/g and results in increased maximal uptake from 0.41 g/g to 0.70 g/g for MOF-303 and MOF-LA2-1, respectively. This small change also influenced the mechanism of adsorption: in MOF-303 water adsorbs in a continuous way, whereas in MOF-LA2-1 water adsorbs through the first order phase transition (Figure S3). Because of that in MOF-LA2-1 we observe vertical jump between low- and high-density states. In turn, NU-1000 has the largest pore volume and maximal uptake of 1.4 cm3/g and 1.59 g/g respectively. Here, water also adsorbs through the first order phase transition, however, the energy barrier between low- and high-density states at equilibrium pressure (step location on isotherm) is much higher in NU-1000 than in in MOF-LA2-1 (420 kJ/mol vs 30 kJ/mol for NU-1000 and MOF-LA2-1 respectively). Among other things, this is reflected in much longer metastable phases. In this manner we explore three systems with different adsorption mechanisms: from continuous adsorption through noncontinuous adsorption with very small energy barrier up to noncontinuous adsorption with large energy barrier (and long metastable regions).

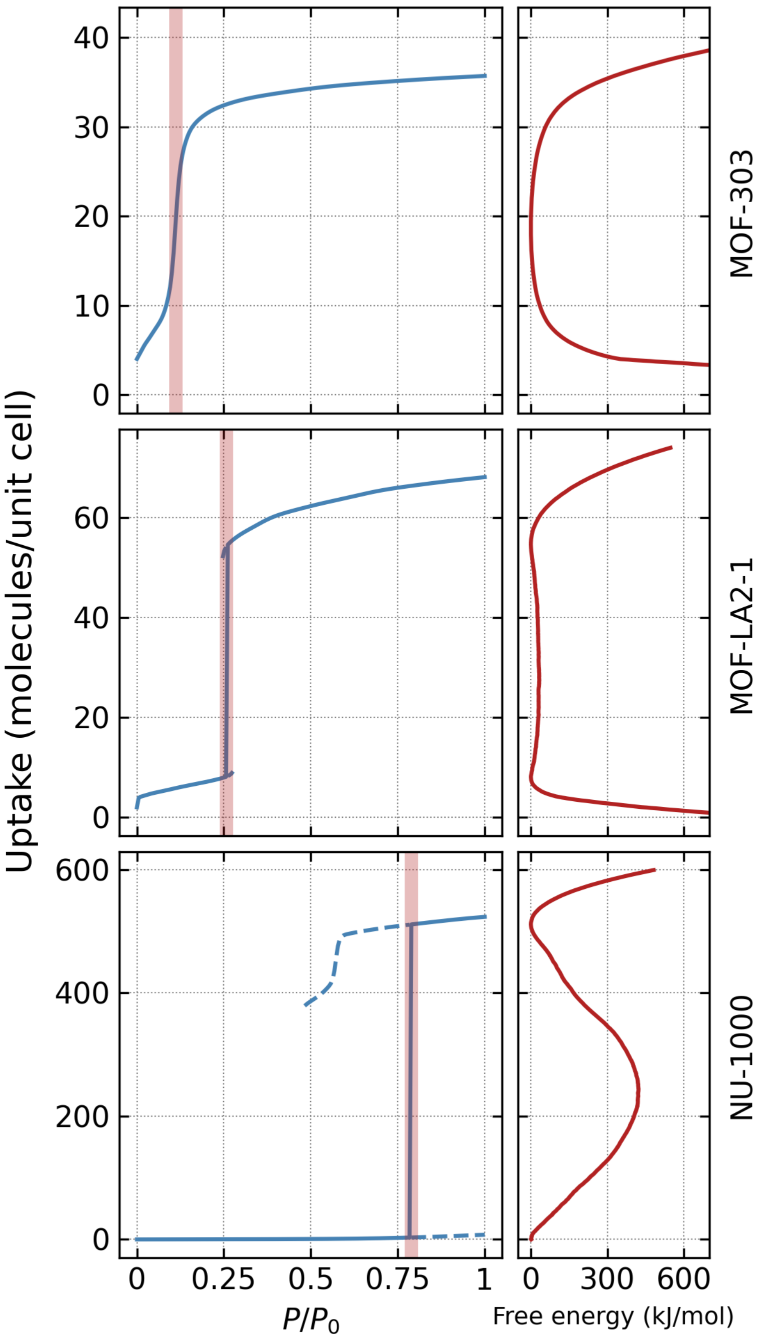

Figure S5. Isotherms of water adsorption at 298 K in (top) MOF-303, (middle) MOF-LA2-1, and (bottom) NU-1000 and corresponding free energy profiles for middle uptake of MOF-303 and phase equilibrium state for MOF-LA2-1 and NU-1000. Solid and dashed lines correspond to the track of stable and metastable states, respectively.

SIMULATION DETAILS

All simulations were performed with our in-house modification of RASPA2 code[5], in which we added ghost swap move, which was already described in the *Methods* section. All *NVT + ghost swap* simulations were conducted for at least 50 000 of initialization cycles and 100 000 production cycles. RASPA cycle contains of $\max\{20, N\}$ number of moves, where N represents the number of fluid molecules in the system. To determine convergence, we monitored the ratio of the probability of accepting insertion and deletion of a molecule (Figure S8). Fugacity coefficient was fixed in all simulations at a value of 1, as we wanted to have full control over chemical potential (μ) value during simulation. During post-processing, we calculated pressure using the fugacity coefficient obtained from WL-TMMC simulations performed with FEASST software[6]. Saturation pressures (P_0) used to calculate P/P_0 at different temperatures were calculated for a TIP4P water model as average from 5 independent simulations. The error was calculated as the standard deviation of the average. The resulting values are provided in Table S1.

Table S1. Saturation pressure values for TIP4P model.

Temperature (K)	Pressure (Pa)	Error (Pa)	Fugacity coefficient
298	4549.85	14.93	0.9792421
303	6098.97	32.94	0.9795284
308	8111.02	56.10	0.9769812
313	10637.44	45.18	0.9742917
318	13763.79	15.68	0.9749976
323	17763.16	37.87	0.9723541
333	28619.65	86.53	0.9686152
343	44821.82	261.19	0.9644947

To model water, we used the TIP4P model[7]. For MOF-303 we used the framework model FF3-E4D4 from the paper of Chheda et al.[8], for MOF-LA2-1 we used the model ZUS(w)-trans,trans form paper by Hanikel et al.[2], and for NU-1000, we adapted the CIF structure from the paper of Mondloch et al.[3], in which we modified the Zr-cluster according to the study by Planas et al.[9] and then optimized the geometry using the plane-wave density functional theory (DFT) method implemented in the Vienna Ab Initio Simulation Package (VASP version 6.3.2)[10–12]. We used the Perdew-Burke-Ernzerhof (PBE) exchange-correlation density functional[13] together with Grimme's D3 dispersion correction with Becke-Johnson damping[14,15]. The lattice cell was fixed during optimization. A plane-wave basis set with an energy cutoff of 520 eV was used and the structure was optimized until the electronic energies and forces on the atoms converged within 10^{-6} eV and 0.02 eV/Å respectively. Only Γ-point was used to sample the Brillouin zone, due to the large unit cell of the structure. Atomic point charges for the force field were calculated using the DDEC6 method[16,17]. For MOF-303 and MOF-LA2-1 force field parameters were adopted from cited works and for NU-1000 we used a combination of parameters from UFF force field[18] for Zr and O atoms and Dreiding force field[19] for remaining atoms. All the frameworks were modelled as rigid. The Lorentz-Berhelot mixing rules were used to

describe interatomic LJ interactions. Interactions were truncated at the spherical cutoff of 12.8 Å and analytical tail corrections were employed for oxygen-oxygen LJ interactions of the water molecule. For electrostatic interactions we used the Ewald summation. To aid in reproducibility, the example simulation files and modified RASPA code are provided in https://github.com/b-mazur/.

The maximum number of molecules (N_{max}) for each MOF was calculated based on the probe-occupiable volume of the framework[20] and the water density at 298 K. The probe-occupiable volume was calculated using Zeo++ software[21], with a spherical helium molecule with a radius of 1.32 Å as a probe and 50000 Monte Carlo samples per unit cell.

WL/TMMC VALIDATION

To validate results obtained with *NVT + ghost swap* method we calculated water adsorption isotherms at 298 K for the three presented systems using a hybrid Wang-Landau and Transition Matrix Monte Carlo method as implemented in FEASST version 0.24.3[6]. The range of the number of particles was divided into windows with a minimum size of 5. The macrostate probability distribution was obtained by stitching together all MPDs from each window. The MC trials consist of trial translation, rotation, and configurational bias insertion/deletion with relative probability of 1:1:4, respectively. The simulation was initialized with translation, rotation and insertion moves until reached required number of molecules. Then the configuration was equilibrated with 10^6 MC trials. The collection matrix began to be populated when Wang-Landau completed 20 flatness checks, and after 25 flatness checks the simulation switched to TMMC. Example simulation files are provided in https://github.com/b-mazur/.

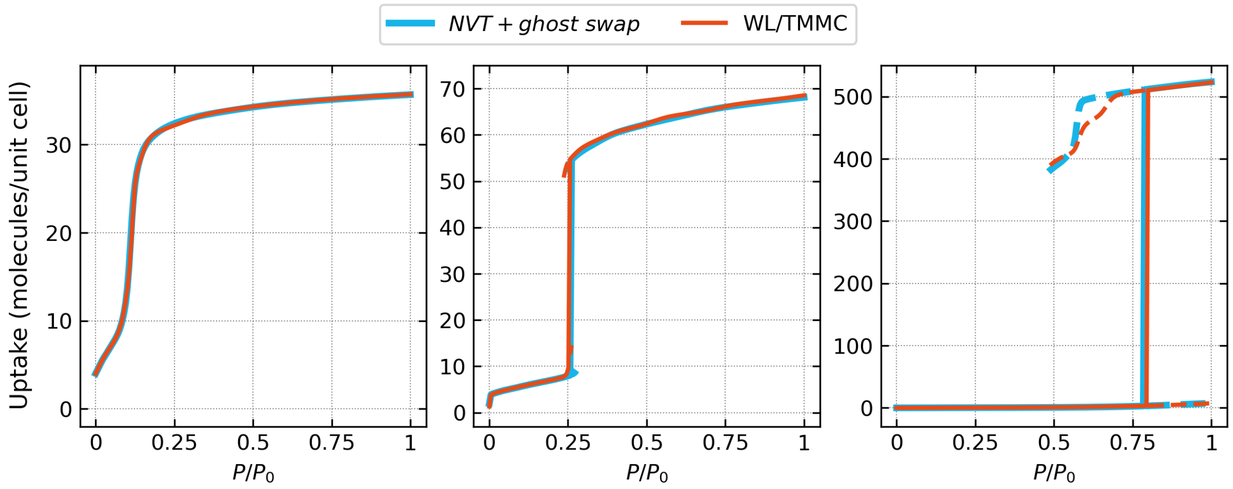

Figure S6. Water adsorption isotherms at 298 K in (left) MOF-303, (middle) MOF-LA2-1 and (right) NU-1000 calculated using (blue) *NVT + ghost swap* method and (red) WL/TMMC method. The trace of the stable phase is plotted with a solid line and that of the metastable phases with a dashed line.

WORKING CAPACITY CALCULATION

The working capacity of NU-1000 was calculated as the difference between the uptake at P/P_0 = 0.7 and P/P_0 = 0.8. In the calculations, we used only the isotherm branch of the stable phase.

Table S2. The working capacity of NU-1000 calculated using different numbers of macrostates. The error was calculated as a reference to the value for N = 1200. Uptake unit is molecules/unit cell.

Macrostates	Uptake (P/P_0 = 0.7)	Uptake (P/P_0 = 0.8)	Working capacity	Error
12	52.45	519.33	466.88	8.51%
24	23.88	515.86	491.98	3.60%
60	9.51	513.92	504.41	1.16%
1200	1.90	512.23	510.33	---

SIMULATION CONVERGENCE

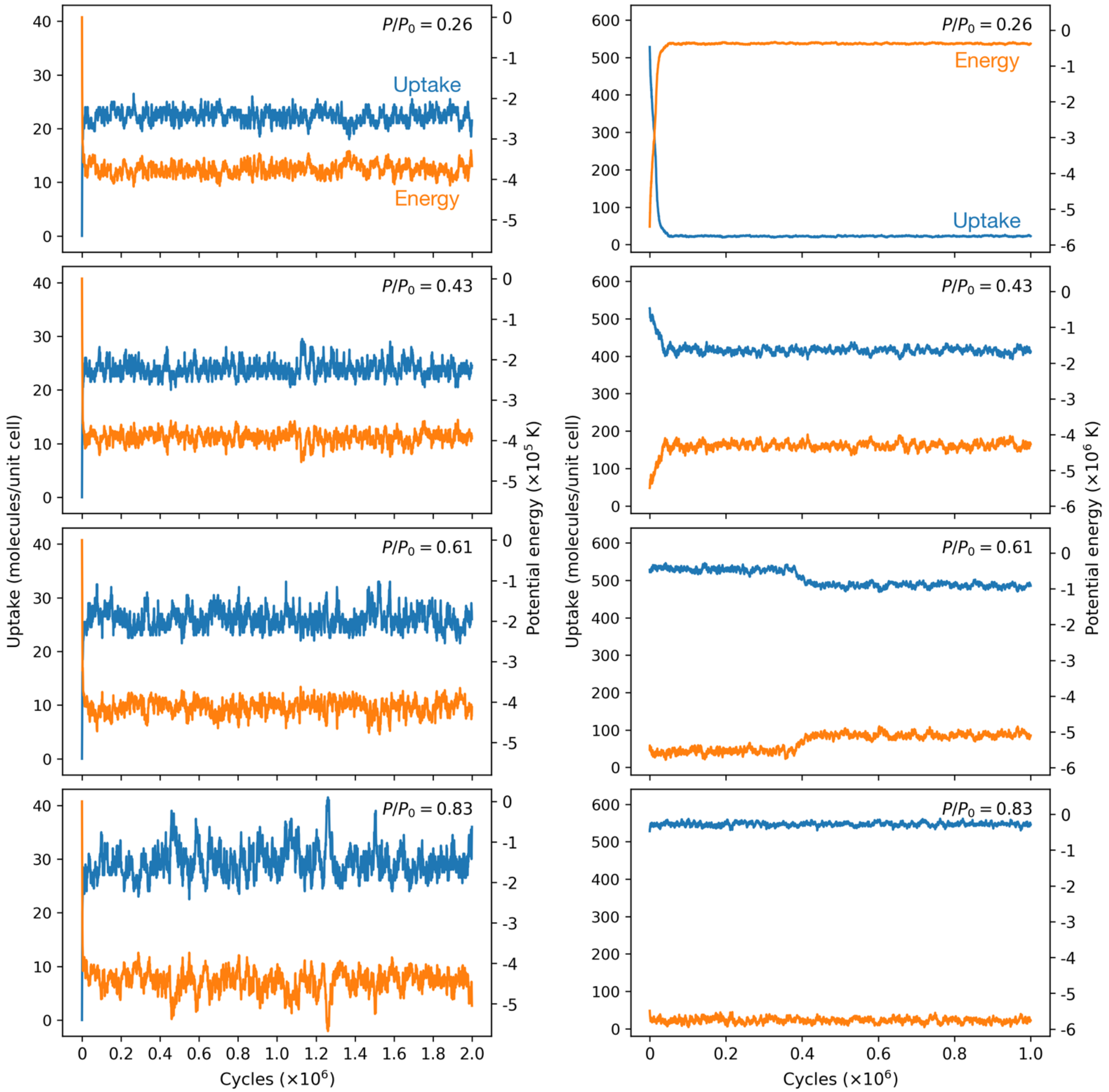

Figure S7. Uptake and potential energy as a function of GCMC simulation cycles for (left) adsorption and (right) desorption of water in NU-1000 at 298 K.

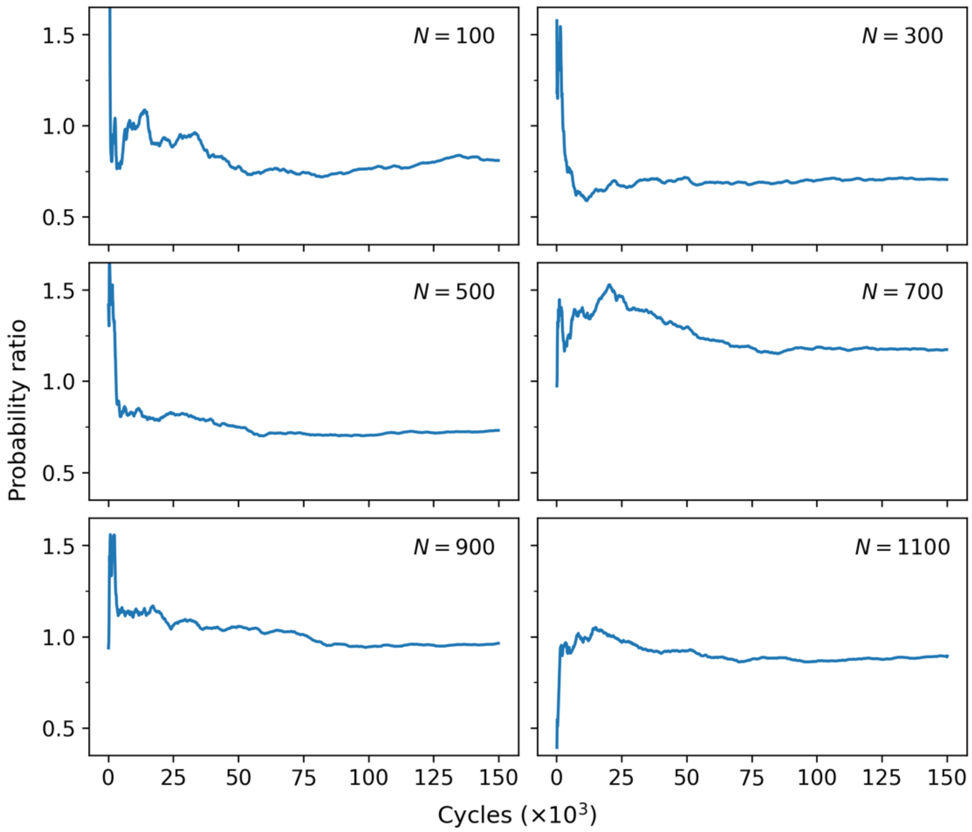

Figure S8. Ratio of probabilities of inserting and deleting molecule at uptake indicated by the value in the top right of each subplot for water adsorption in NU-1000 at 298 K.

COMPARISON WITH EXPERIMENTAL DATA

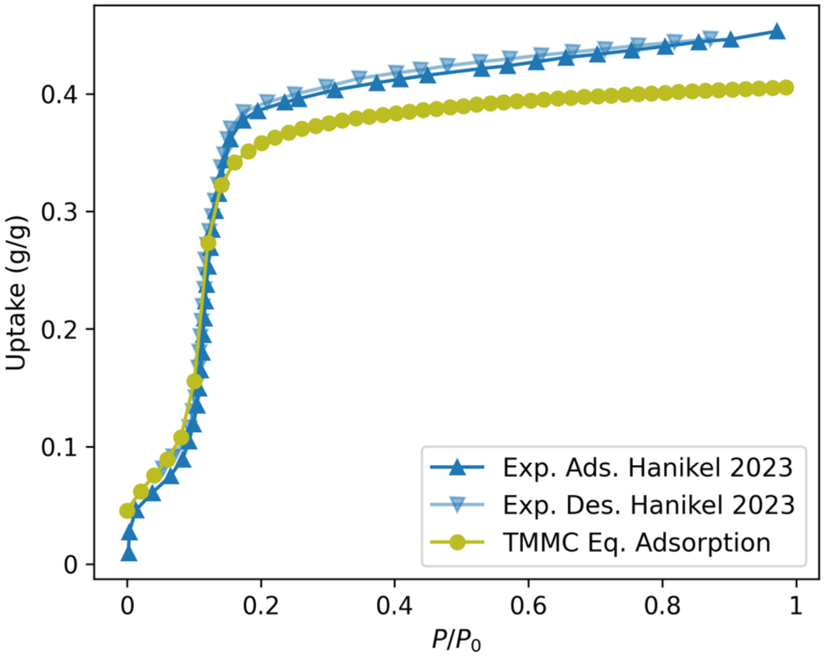

Figure S9. Experimental[2] (blue) and simulated using TMMC (yellow) water adsorption isotherms in MOF-303 at 298 K. The experimental data was obtained through digitization of Figure 2b from the cited paper.

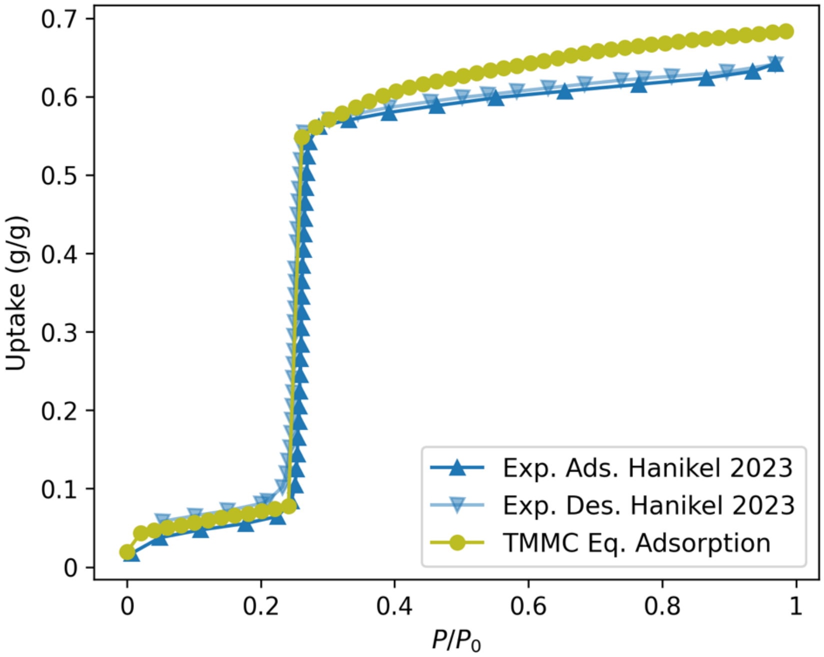

Figure S10. Experimental[2] (blue) and simulated using TMMC (yellow) water adsorption isotherms in MOF-LA2-1 at 298 K. The experimental data was obtained through digitization of Figure 2b from the cited paper.

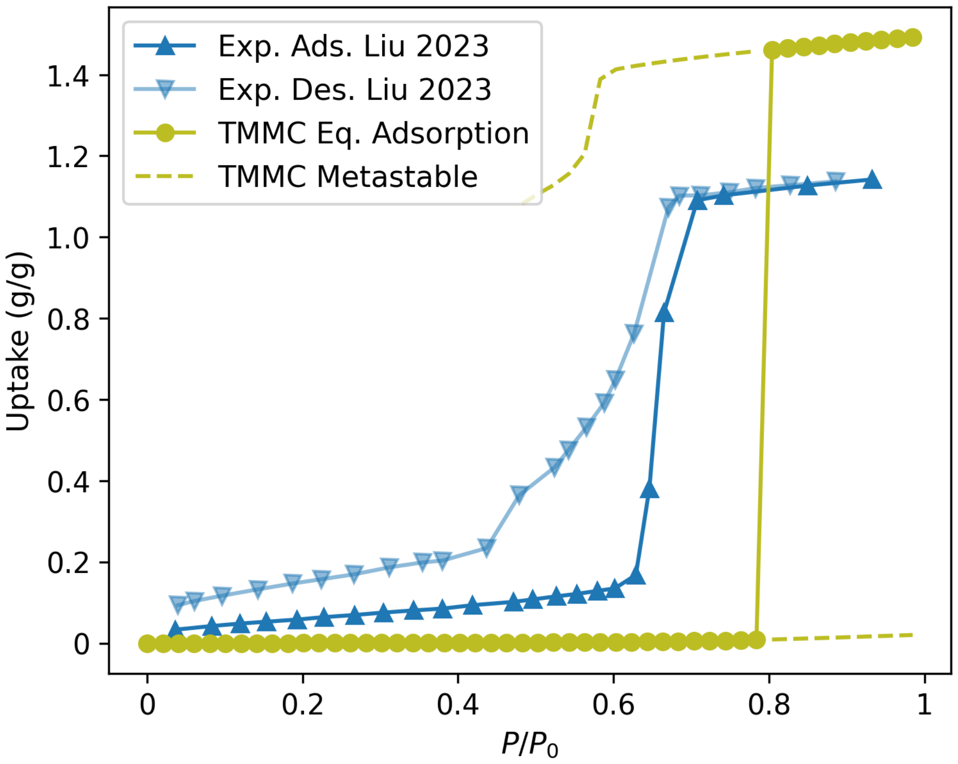

Figure S11. Experimental[22] (blue) and simulated using TMMC (yellow) water adsorption isotherms in NU-1000 at 298 K. Trace of metastable states is marked with yellow dashed line. The experimental data was obtained through digitization of Figure 2a from the cited paper.

FREE ENERGY MAP

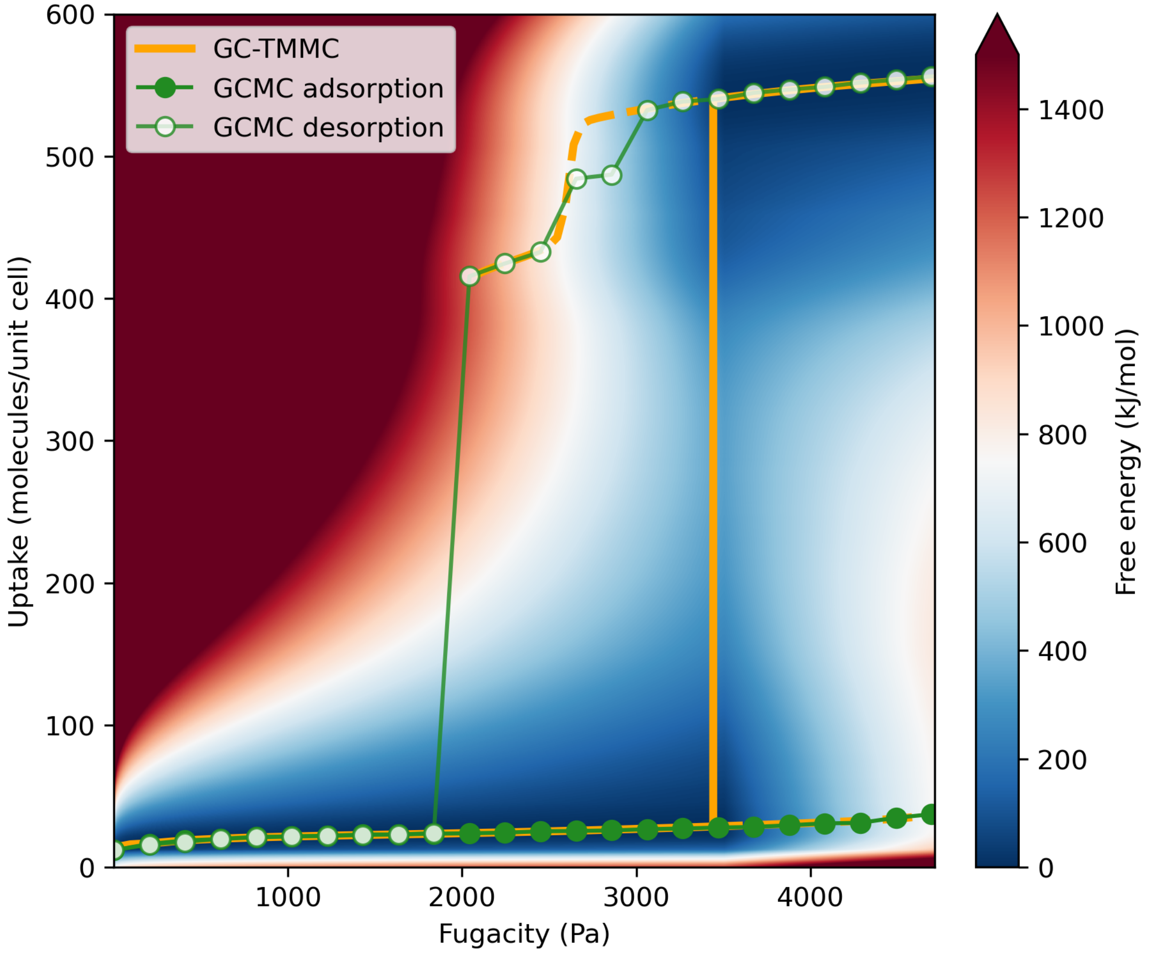

Figure S12. Map of the free energy of water adsorption in NU-1000 at 298 K as a function of fugacity and uptake. Isotherm calculated with GC-TMMC is displayed with yellow line and isotherms calculated with GCMC are marked with green full symbols for adsorption and green empty symbols for desorption.

REFERENCES

(1) Fathieh, F.; Kalmutzki, M. J.; Kapustin, E. A.; Waller, P. J.; Yang, J.; Yaghi, O. M. Practical Water Production from Desert Air. *Sci Adv* **2018**, *4* (6). https://doi.org/10.1126/sciadv.aat3198.

(2) Hanikel, N.; Kurandina, D.; Chheda, S.; Zheng, Z.; Rong, Z.; Neumann, S. E.; Sauer, J.; Siepmann, J. I.; Gagliardi, L.; Yaghi, O. M. MOF Linker Extension Strategy for Enhanced Atmospheric Water Harvesting. *ACS Cent Sci* **2023**, *9* (3), 551–557. https://doi.org/10.1021/acscentsci.3c00018.

(3) Mondloch, J. E.; Bury, W.; Fairen-Jimenez, D.; Kwon, S.; DeMarco, E. J.; Weston, M. H.; Sarjeant, A. A.; Nguyen, S. T.; Stair, P. C.; Snurr, R. Q.; Farha, O. K.; Hupp, J. T. Vapor-Phase Metalation by Atomic Layer Deposition in a Metal–Organic Framework. *J Am Chem Soc* **2013**, *135* (28), 10294–10297. https://doi.org/10.1021/ja4050828.

(4) Liu, X.; Wang, X.; Kapteijn, F. Water and Metal-Organic Frameworks: From Interaction toward Utilization. *Chem Rev* **2020**, *120* (16), 8303–8377. https://doi.org/10.1021/ACS.CHEMREV.9B00746/ASSET/IMAGES/LARGE/CR9B00746_0019.JPEG.

(5) Dubbeldam, D.; Calero, S.; Ellis, D. E.; Snurr, R. Q. RASPA: Molecular Simulation Software for Adsorption and Diffusion in Flexible Nanoporous Materials. *Mol Simul* **2016**, *42* (2), 81–101. https://doi.org/10.1080/08927022.2015.1010082.

(6) Hatch, H. W.; Mahynski, N. A.; Shen, V. K. FEASST: Free Energy and Advanced Sampling Simulation Toolkit. *J Res Natl Inst Stand Technol* **2018**, *123*, 123004. https://doi.org/10.6028/jres.123.004.

(7) Jorgensen, W. L.; Chandrasekhar, J.; Madura, J. D.; Impey, R. W.; Klein, M. L. Comparison of Simple Potential Functions for Simulating Liquid Water. *J Chem Phys* **1983**, *79* (2), 926–935. https://doi.org/10.1063/1.445869.

(8) Chheda, S.; Jeong, W.; Hanikel, N.; Gagliardi, L.; Siepmann, J. I. Monte Carlo Simulations of Water Adsorption in Aluminum Oxide Rod-Based Metal–Organic Frameworks. *The Journal of Physical Chemistry C* **2023**, *127* (16), 7837–7851. https://doi.org/10.1021/acs.jpcc.3c00354.

(9) Planas, N.; Mondloch, J. E.; Tussupbayev, S.; Borycz, J.; Gagliardi, L.; Hupp, J. T.; Farha, O. K.; Cramer, C. J. Defining the Proton Topology of the Zr_6 -Based Metal–Organic Framework NU-1000. *J Phys Chem Lett* **2014**, *5* (21), 3716–3723. https://doi.org/10.1021/jz501899j.

(10) Kresse, G.; Hafner, J. *Ab Initio* Molecular Dynamics for Liquid Metals. *Phys Rev B* **1993**, *47* (1), 558–561. https://doi.org/10.1103/PhysRevB.47.558.

(11) Kresse, G.; Furthmüller, J. Efficiency of Ab-Initio Total Energy Calculations for Metals and Semiconductors Using a Plane-Wave Basis Set. *Comput Mater Sci* **1996**, *6* (1), 15–50. https://doi.org/10.1016/0927-0256(96)00008-0.

(12) Kresse, G.; Furthmüller, J. Efficient Iterative Schemes for *Ab Initio* Total-Energy Calculations Using a Plane-Wave Basis Set. *Phys Rev B* **1996**, *54* (16), 11169–11186. https://doi.org/10.1103/PhysRevB.54.11169.

(13) Perdew, J. P.; Burke, K.; Ernzerhof, M. Generalized Gradient Approximation Made Simple. *Phys Rev Lett* **1996**, *77* (18), 3865–3868. https://doi.org/10.1103/PhysRevLett.77.3865.
(14) Grimme, S.; Antony, J.; Ehrlich, S.; Krieg, H. A Consistent and Accurate *Ab Initio* Parametrization of Density Functional Dispersion Correction (DFT-D) for the 94 Elements H-Pu. *J Chem Phys* **2010**, *132* (15). https://doi.org/10.1063/1.3382344.
(15) Grimme, S.; Ehrlich, S.; Goerigk, L. Effect of the Damping Function in Dispersion Corrected Density Functional Theory. *J Comput Chem* **2011**, *32* (7), 1456–1465. https://doi.org/10.1002/jcc.21759.
(16) Manz, T. A.; Limas, N. G. Introducing DDEC6 Atomic Population Analysis: Part 1. Charge Partitioning Theory and Methodology. *RSC Adv* **2016**, *6* (53), 47771–47801. https://doi.org/10.1039/C6RA04656H.
(17) Limas, N. G.; Manz, T. A. Introducing DDEC6 Atomic Population Analysis: Part 2. Computed Results for a Wide Range of Periodic and Nonperiodic Materials. *RSC Adv* **2016**, *6* (51), 45727–45747. https://doi.org/10.1039/C6RA05507A.
(18) Rappe, A. K.; Casewit, C. J.; Colwell, K. S.; Goddard, W. A.; Skiff, W. M. UFF, a Full Periodic Table Force Field for Molecular Mechanics and Molecular Dynamics Simulations. *J Am Chem Soc* **1992**, *114* (25), 10024–10035. https://doi.org/10.1021/ja00051a040.
(19) Mayo, S. L.; Olafson, B. D.; Goddard, W. A. DREIDING: A Generic Force Field for Molecular Simulations. *J Phys Chem* **1990**, *94* (26), 8897–8909. https://doi.org/10.1021/j100389a010.
(20) Ongari, D.; Boyd, P. G.; Barthel, S.; Witman, M.; Haranczyk, M.; Smit, B. Accurate Characterization of the Pore Volume in Microporous Crystalline Materials. *Langmuir* **2017**, *33* (51), 14529–14538. https://doi.org/10.1021/acs.langmuir.7b01682.
(21) Willems, T. F.; Rycroft, C. H.; Kazi, M.; Meza, J. C.; Haranczyk, M. Algorithms and Tools for High-Throughput Geometry-Based Analysis of Crystalline Porous Materials. *Microporous and Mesoporous Materials* **2012**, *149* (1), 134–141. https://doi.org/10.1016/j.micromeso.2011.08.020.
(22) Liu, J.; Prelesnik, J. L.; Patel, R.; Kramar, B. V.; Wang, R.; Malliakas, C. D.; Chen, L. X.; Siepmann, J. I.; Hupp, J. T. A Nanocavitation Approach to Understanding Water Capture, Water Release, and Framework Physical Stability in Hierarchically Porous MOFs. *J Am Chem Soc* **2023**, *145* (51), 27975–27983. https://doi.org/10.1021/jacs.3c07624.
